# Effects of critical interfacial shear strength between a polymer matrix and carbon nanotubes on the interphase strength and Pukanszky's “*B*” interphase parameter

**DOI:** 10.1039/d0ra00978d

**Published:** 2020-04-03

**Authors:** Yasser Zare, Kyong Yop Rhee

**Affiliations:** Department of Mechanical Engineering, College of Engineering, Kyung Hee University 1 Seocheon, Giheung Yongin 446-701 Gyeonggi Republic of Korea rheeky@khu.ac.kr +82 31 202 6693 +82 31 201 2565

## Abstract

In this paper, the “*B*” interphase parameter in the Pukanszky model and interphase strength for polymer carbon nanotube (CNT) nanocomposites are expressed by the critical interfacial shear strength (*τ*_c_) and interfacial shear strength (*τ*) between a polymer matrix and CNTs. A suggested model and a developed Pukanszky model for tensile strength of nanocomposites are combined to develop the equations for “*B*” and interphase strength. Many experimental data for various samples confirm the models. The impacts of all parameters on the “*B*” and interphase strength are explained to approve the developed equations. The contour plots display the same trends for the roles of all parameters in the “*B*” and interphase strength. Low “*τ*_c_”, high “*τ*”, thin and large CNTs as well as a dense interphase are ideal to obtain the high levels for “*B*” and interphase strength. Among the studied parameters, CNT size largely controls the “*B*” and interphase strength, while the waviness and strength of CNTs play insignificant roles.

## Abstract 2

In this paper, the “*B*” interphase parameter in the Pukanszky model and interphase strength for polymer carbon nanotube (CNT) nanocomposites are expressed by the critical interfacial shear strength (*τ*_c_) and interfacial shear strength (*τ*) between a polymer matrix and CNTs.

## Graphical abstract

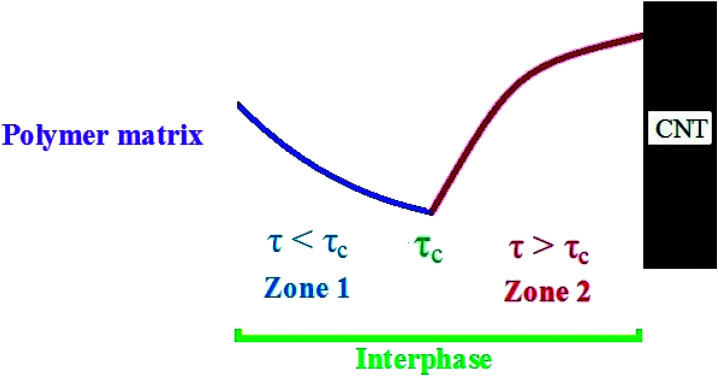

## 1 Introduction

Carbon nanotubes (CNT) can noticeably increase the performance of nanocomposites, because they include high aspect ratio, very high modulus and considerable conductivity.^1–10^ The high aspect ratio of CNTs produces a low percolation threshold in nanocomposites establishing a CNT network by a low concentration of CNTs.^11,12^ Accordingly, polymer CNT nanocomposites are good candidates for many applications such as electronics, biosensors, actuators, aerospace structures and automotive components.^13–21^ The main difficulties for the production of polymer CNT nanocomposites include the deprived spreading of CNTs and the poor interfacial attachment between polymer media and CNTs.^22,23^ As a result, improving the processing terms and using the modified/functionalized nanoparticles are recommended to solve these problems and promote the nanocomposite performance.^24–35^

The interphase region between polymer matrix and nanoparticles plays an important reinforcing efficiency in nanocomposites.^36–41^ Moreover, the interphase region joins to the network in nanocomposites improving the mechanical properties and electrical conductivity.^42–45^ In fact, a thicker interphase causes a lower percolation threshold, which enlarges the CNT network and positively manipulates the stress and charge transferring through nanocomposites. These are many modeling work in the literature investigating the interphase characteristics and their roles in the performance of nanocomposites.^46,47^ The modeling techniques are interesting, because the investigational handling of interphase region is very tough due to the nanoscale manipulation, while the models give much information by simple approaches.

The functioning stress transferring among polymer media and nanoparticles is critical to promote the mechanical possessions, since a deprived interphase causes the debonding of nanoparticles from polymer medium during loading, whereas a robust interphase can stand a large volume of stress. Consequently, a potent interphase amplifies the stress bearing promoting the performance of nanocomposites. These observations demonstrate the important roles of interfacial/interphase properties in the nanocomposites. The incomplete interphase unsuccessfully transports the stress from polymer matrix to nanoparticles, because it is not strong enough for efficient stress transferring. In this case, there is a critical interfacial shear strength (*τ*_c_) controlling the stress transferring *via* interphase region. When the interfacial shear strength (*τ*) is poorer than “*τ*_c_”, the interphase cannot provide the reinforcement, but the effective strengthening of nanocomposites by interphase happens when “*τ*” is more than “*τ*_c_”. Thus, “*τ*_c_” plays an important role in the strengthening of polymer nanocomposites, although the previous papers have ignored it.

Many investigators have studied the interphase features in the mechanical presentations of polymer nanocomposites,^47,48^ but they ignored the least level of interfacial shear strength (*τ*_c_), which reinforces the nanocomposites. Actually, the former reports have studied the characteristics and roles of interphase area in the nanocomposites, but they neglected the least interphase terms strengthening the polymer media. The current study tries to express the “*B*” interphase term (in Pukanszky model) and interphase strength in polymer CNT nanocomposites assuming “*τ*_c_” and “*τ*”. At the first step, “*τ*_c_” is defined and its roles in the effective interphase thickness and operative CNT concentration are highlighted. After that, a suggested model for tensile strength of polymer CNT nanocomposites is expressed and the Pukanszky model is developed by “*τ*” and “*τ*_c_” terms. Many experimental data are applied to confirm the models. These models are joined to express the “*B*” term in Pukanszky model as a function of “*τ*”, “*τ*_c_” and CNT size. Finally, an equation is developed for the interphase strength by “*τ*”, “*τ*_c_”, CNT size and interphase thickness. The roles of all parameters in the “*B*” and interphase strength are explained to confirm the established equations.

## 2 Development of equations

**Fig. 1:**
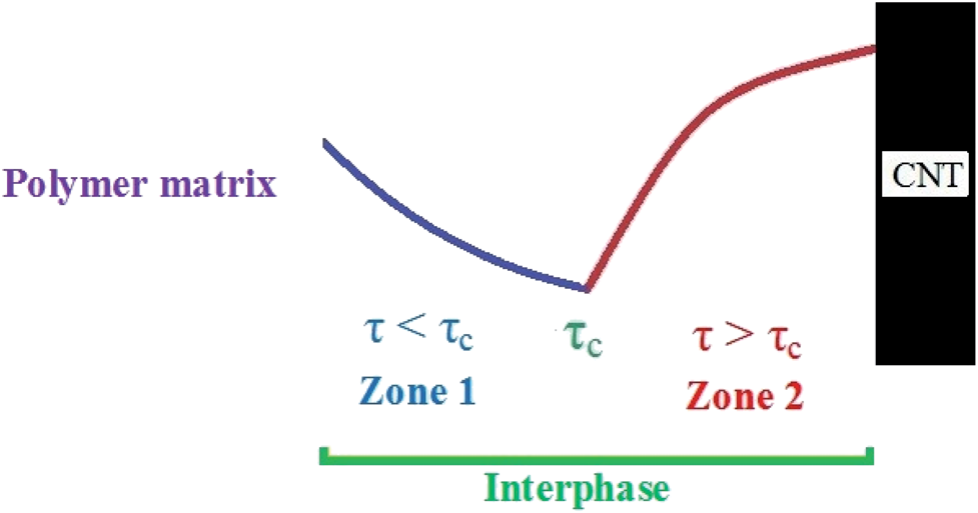
The schematic profile of stress in the interphase region between polymer matrix and CNT: zone 1 (*τ* < *τ*_c_) and zone 2 (*τ* > *τ*_c_).

In the case of imperfect interphase between polymer matrix and CNT, which is normal in nanocomposites, the interphase region is not strong enough for efficient stress transferring from polymer matrix to CNT. In this condition, a critical level for interfacial shear strength (*τ*_c_) exists, which manipulates the stress transferring between polymer matrix and nanoparticles. Fig. 1 presents the profile of stress in the interphase region surrounding CNT. At *τ* < *τ*_c_ (zone 1), the stress decreases, because it's far from the stiff CNT and the interphase is very weak in this region. Nevertheless, when “*τ*” exceeds the “*τ*_c_”, the interphase is strong enough to stand the stress transferred from polymer matrix to CNT causing the reinforcement of nanocomposites.

“*τ*_c_” as the least interfacial shear strength tolerating the loaded stress from polymer matrix to CNT is defined as:1
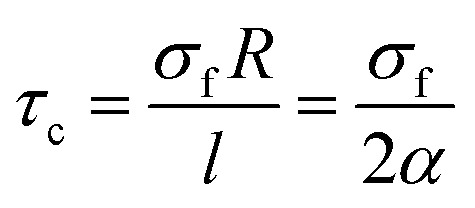
where “*σ*_f_”, “*R*” and “*l*” are the strength, radius and length of CNT, respectively and “*α*” shows the aspect ratio as *α* = *l*/*d* (*d* is CNT diameter).

We derived this equation by the definition of “*L*_c_” as the least length of CNT for effective transferring of stress from polymer matrix to nanoparticles 
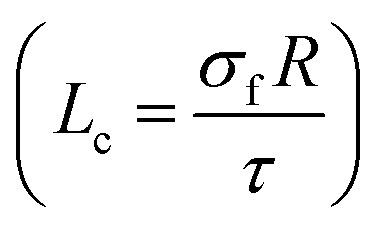
.^49^ According to “*L*_c_” equation, the critical interfacial shear strength is defined by the properties of CNT. Accordingly, “*τ*_c_” does not depend on the interphase properties, explicitly, but “*τ*_c_” correlates to the CNT aspect ratio controlling the extent of interfacial area between polymer matrix and CNT. Actually, “*τ*_c_” indirectly correlates to the interfacial/interphase properties in nanocomposites.

The significant length of CNT makes the waviness in nanocomposites.^50^ The effective length of CNT (*l*_eff_) is the smallest distance between two ends of curved CNT defining the waviness parameter as:2
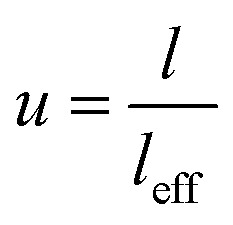
where *u* = 1 displays the straight CNT, but a higher “*u*” reveals more waviness.

When “*l*_eff_” is considered into eqn (1), “*τ*_c_” is developed to:3
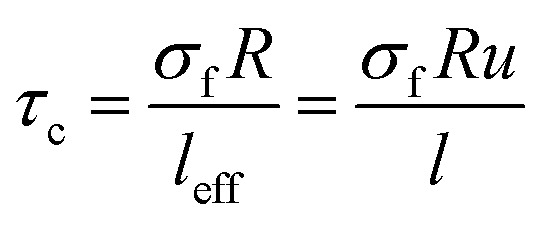


As mentioned, the properties of interphase region in nanocomposites correlate to the values of “*τ*_c_” and “*τ*”. Therefore, the effective interphase thickness is expressed at two zones (Fig. 1) as:4
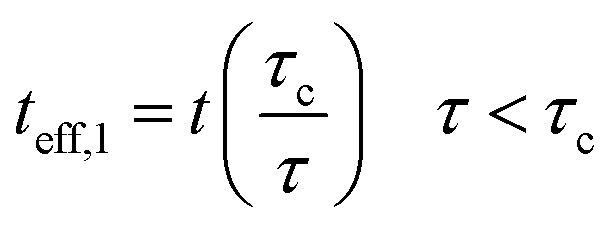
5
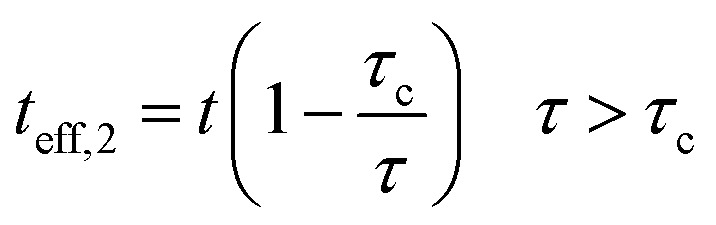
where “*t*” is interphase thickness. The contributions of both zones suggest the effective interphase thickness as:6
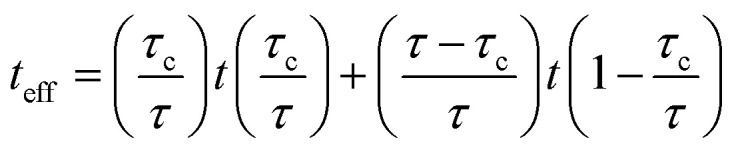


Additionally, the effective volume fraction of CNT in the nanocomposites comprises both CNT and interphase portions^51^ as:7
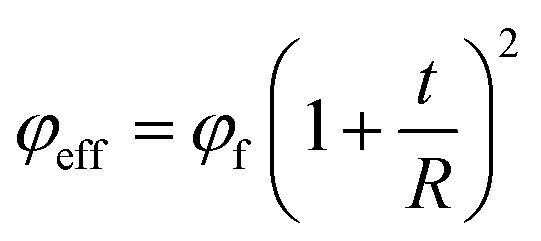
where “*φ*_f_” is CNT volume fraction. When “*t*_eff_” is replaced from eqn (6) into the latter equation, “*φ*_eff_” can consider the effective interphase thickness using “*τ*_c_” and “*τ*”.

The former studies established that the tensile strength of nanocomposites directly correlates to polymer strength (*σ*_m_), an orientation factor (*η*_o_), “*τ*”, “*α*” and “*φ*_f_”^52,53^ as:

8 *σ* ≈ *σ*_m_ + *η*_o_*ταφ*_f_

Assuming the roles of “*φ*_eff_” and *τ* − *τ*_c_, the latter equation was developed in our previous article (submitted one) as:9
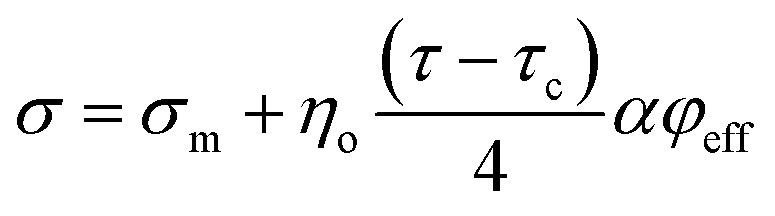
suggesting the relative strength of nanocomposites as *σ*_R_ = *σ*/*σ*_m_ by *η*_o_ = 0.2 (ref. 52) as:10
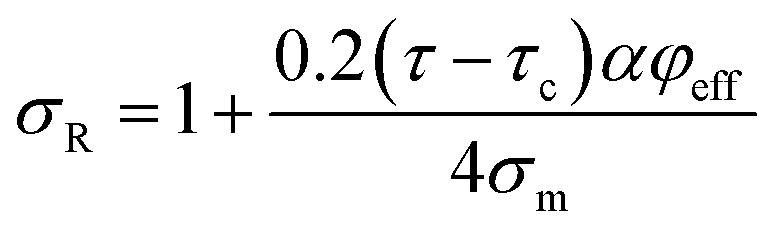


Pukanszky^54^ developed a simple model for tensile strength of nanocomposites assuming the interphase properties as:11
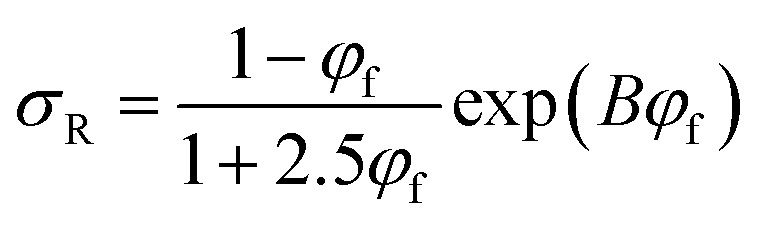
where “*B*” is an interphase parameter, which demonstrates the extent of interphase properties between polymer and nanoparticles. “*B*” is defined as:12
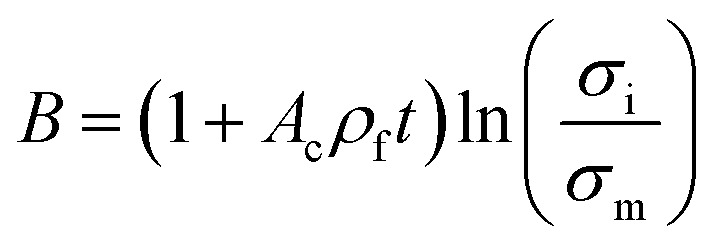
where “*A*_c_” is the specific surface area of nanofiller, “*ρ*_f_” is filler density and “*σ*_i_” denotes the strength of interphase region.

The Pukanszky model can be developed assuming “*φ*_eff_” as:13
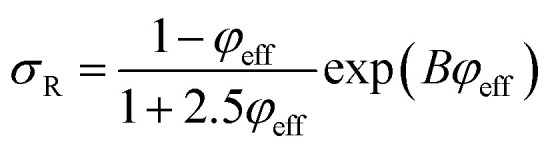


The developed Pukanszky model can be rearranged to:14
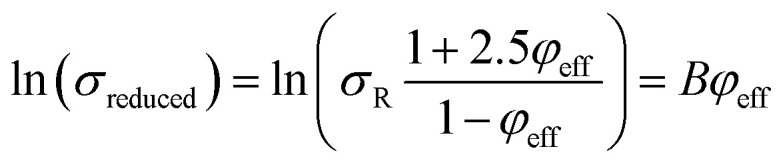
where the linear correlation between ln(*σ*_reduced_) and “*φ*_eff_” gives the “*B*” slope.

The specific surface area of CNT as the surface area (*A*) per mass (*m*) can be expressed by:15
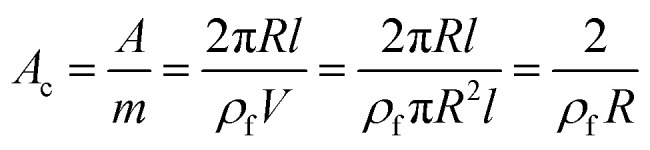
where “*V*” is the volume of CNT.

By substituting of “*A*_c_” from the latter equation into eqn (12), “*B*” is obtained as:16
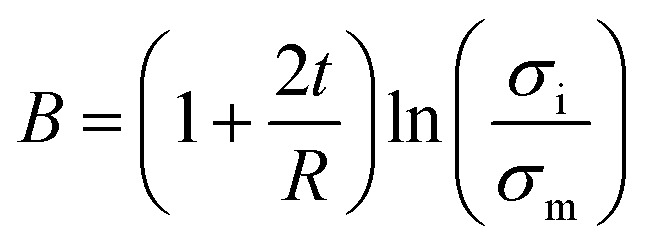


Now, the suggested equation (eqn (10)) and the developed Pukanszky model (eqn (13)) are joined to express the “*B*” and “*σ*_i_” terms by “*τ*_c_” and “*τ*”.

Eqn (14) can be rewritten as:17
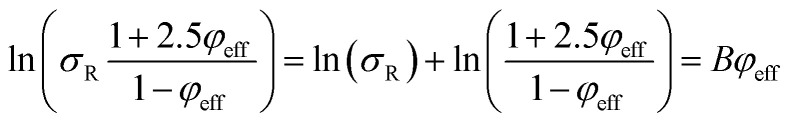


Substituting of “*σ*_R_” from eqn (10) into the latter equation expresses:18
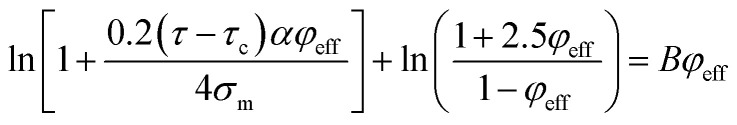


At very low “*φ*_eff_” (*φ*_eff_ ≪ 1), it is approximated that:19
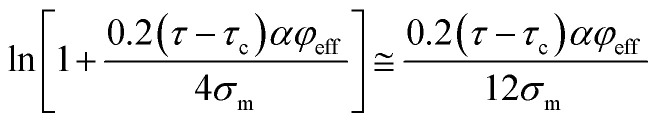
20
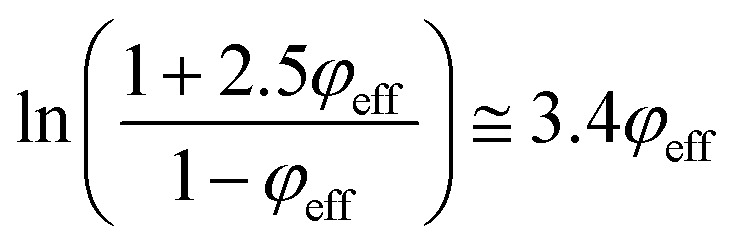


When eqn (19) and (20) are replaced into eqn (18), the following equation is obtained as:21
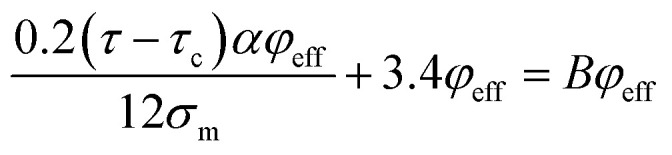
expressing the “*B*” term as a function of “*τ*_c_” and “*τ*” as:22
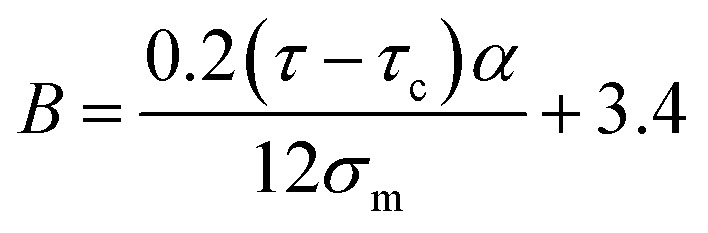


Moreover, when “*B*” is replaced from eqn (16) into the latter equation, “*σ*_i_” is given by:23
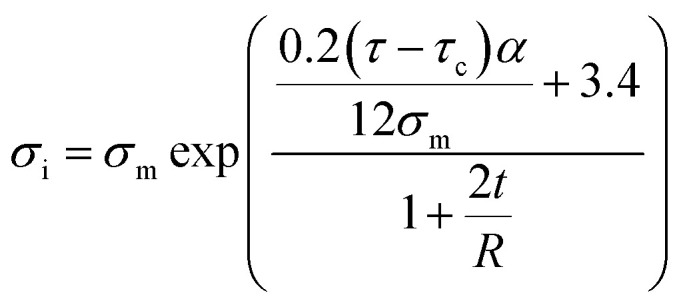
correlating the interphase strength to “*τ*_c_”, “*τ*”, CNT size and interphase thickness.

**Fig. 2:**
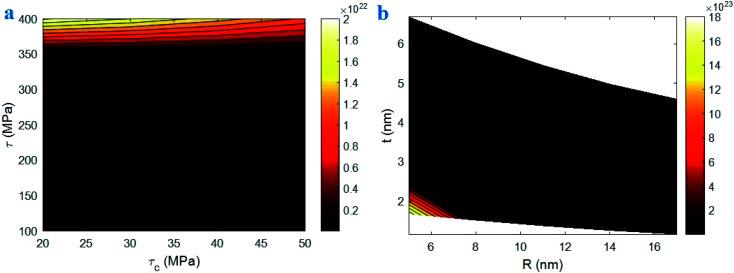
Correlations of interphase strength to (a) “*τ*_c_” and “*τ*” and (b) “*R*” and “*t*” parameters using eqn (23).

Fig. 2a exhibits the contour plot for the roles of “*τ*_c_” and “*τ*” parameters in the interphase strength at average *σ*_m_ = 30 MPa, *R* = 10 nm, *u* = 1.2, *l* = 10 μm and *t* = 5 nm. It is observed that the interphase strength reaches to 2 × 10^22^ MPa at *τ*_c_ = 20 MPa and *τ* = 400 MPa. However, this level is not reasonable, because the interphase strength cannot surpass the CNT strength as average 30 GPa.^46^ Therefore, eqn (23) gives the inaccurate levels for interphase strength.

In addition, Fig. 2b shows the calculations of interphase strength at different ranges of “*R*” and “*t*” and average *τ* = 200 MPa. The interphase strength considerably increases to 18 × 10^23^ MPa at *R* = 5 nm and *t* = 1.7 nm. This level for interphase strength is not reasonable, as mentioned. It is also shown that the interphase strength improves by thin interphase indicating the inverse relation between interphase strength and thickness, while both interphase thickness and strength directly correlates to the extents of interfacial interaction/adhesion between polymer matrix and nanoparticles.^55,56^ So, eqn (23) is not appropriate for the interphase strength in CNT nanocomposites.

It can be concluded that the exp function considerably grows the interphase strength based on eqn (23). Also, eqn (23) wrongly shows the relationship between interphase strength and thickness. Therefore, eqn (23) can be modified by deleting exp function as:24
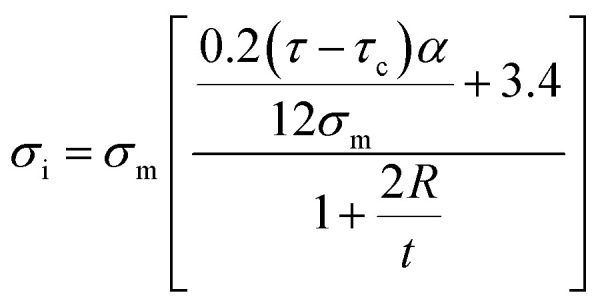
which presents the interphase strength in CNT nanocomposites as a function of polymer strength, “*τ*_c_”, “*τ*”, CNT dimensions and interphase thickness. When we have a distribution of each parameter, we apply the average level of each parameter in the equations. In fact, when the different levels of parameters are available, we consider the average and reasonable range for each factor.

The mentioned equations have some limits. These equations are only applicable for CNT reinforced nanocomposites or nanocomposites containing cylindrical nanoparticles, because the defined terms including “*τ*_c_”, “*u*”, “*φ*_eff_” and “*A*_c_” were defined for the reinforced nanocomposites by cylindrical nanoparticles such as CNT. However, the developed equations by Lazzeri and Phuong^49^ are applicable for polymer nanocomposites and composites comprising clay, CNT and wood flour. Moreover, our developed equations are valid for the large aspect ratio of CNT. Also, *t* = 0 causes the meaningless value for “*σ*_i_” (eqn (24)); so, the developed equations are applicable when the samples include the interphase area (*t* > 0). In addition, it is crucial to obtain the fine dispersion of CNT in the polymer matrix, because the aggregation/agglomeration of nanoparticles restricts the interfacial area and shortens the filler aspect ratio.^57,58^

## 3 Results and discussion

### 3.1 Confirmation of models

The experimental data of relative strength for several samples can validate the suggested model (eqn (10)) and the developed Pukanszky model (eqn (14)). In other words, the appropriate agreements between experimental results and predictions confirm the models. Four samples including polysilsesquioxane/multi-walled CNT (MWCNT),^59^ chitosan-*g*-MWCNT,^60^ polyacrylonitrile (PAN)/MWCNT nanofiber^61^ and chitosan/MWCNT^62^ were chosen from the valid papers. Table 1 shows the details for the samples from the original references. The values of “*σ*_m_” for the samples were reported from the mentioned references, but some papers did not report the CNT size. We considered the average *R* = 10 nm and *l* = 10 μm for the samples excluding the data of CNT size. Also, the interfacial/interphase properties are calculated using the developed equations in this paper. The average values of “*σ*_f_” and “*u*” are considered as 30 GPa and 1.2, respectively. These data were applied into eqn (10) to estimate the tensile strength of the samples in our previous paper (submitted one). It was observed that all calculations suitably agree with the experimental results confirming the suggested model. As a result, the suggested model can approximate the tensile strength of polymer CNT nanocomposites assuming “*τ*_c_”, “*τ*”, CNT size and interphase thickness.

**Table 1:** Estimations of various parameters for the samples using the models

Samples	*σ*_m_ (MPa)	*R* (nm)	*l* (μm)	*t* (nm)	*τ*_c_ (MPa)	*τ* (MPa)	*t*_eff_ (nm)	*B*	*σ*_i_ (MPa)
Polysilsesquioxane/MWCNT^59^	6.00	10	10	4	36	140	2.50	123.8	81.7
Chitosan-*g*-MWCNT^60^	39.6	8	7.5	10	38.4	223	7.15	33.7	412.7
PAN/MWCNT^61^	70.0	10	10	8	36	217	5.80	21.4	335.4
Chitosan/MWCNT^62^	11.6	10	10	4	36	165	2.60	80.6	108.9

The calculations of various parameters for the samples by the suggested model are given in Table 1. The interphase thickness is calculated from 4 to 10 nm representing the different ranges of interfacial adhesion between polymer matrices and MWCNT. “*τ*_c_” is obtained as 36 and 38.4 MPa for the samples. According to eqn (1), “*τ*_c_” depends on the “*σ*_f_” and CNT size and thus three samples show the same “*τ*_c_”. Additionally, the highest and the lowest levels of “*τ*” are reported for chitosan-*g*-MWCNT and polysilsesquioxane/MWCNT samples, respectively. “*τ*” results for the samples are higher than “*τ*_c_” expressing that the interphase region effectively transfers the stress from polymer matrix to CNT. “*t*_eff_” (eqn (6)) also varies from 2.5 to 7.15 nm, due to the dissimilar ranges of “*τ*_c_” and “*τ*” for the samples. Generally, a higher level of “*τ*” produces a higher “*t*_eff_”, because it raises the efficiency of interphase for stress transferring.

**Fig. 3:**
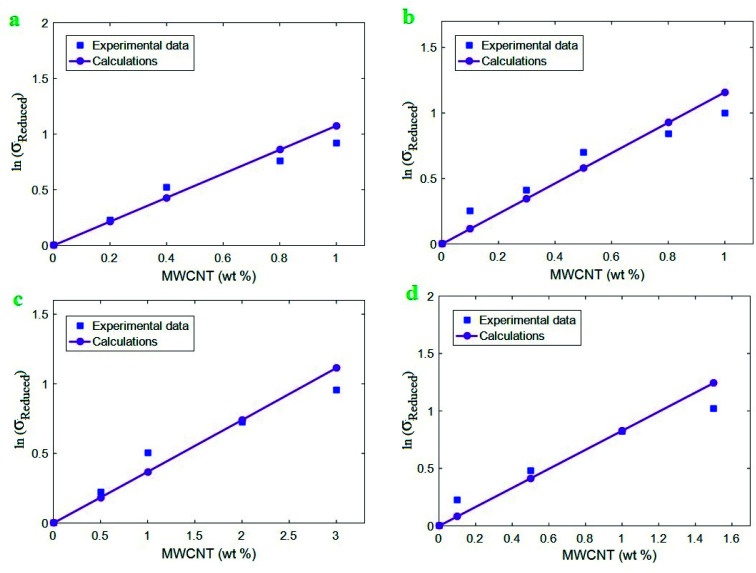
Experimental levels and calculations of ln(*σ*_reduced_) at different CNT concentrations based on eqn (14) for (a) polysilsesquioxane/MWCNT,^59^ (b) chitosan-*g*-MWCNT,^60^ (c) PAN/MWCNT^61^ and (d) chitosan/MWCNT^62^ samples.

The developed Pukanszky model is validated by comparing the experimental and theoretical levels of ln(*σ*_reduced_) at different effective CNT concentrations using eqn (14), as shown in Fig. 3. The calculations properly follow the experimental data at all CNT fractions validating the developed Pukanszky model. By this comparison, it is possible to calculate the “*B*” (eqn (22)) and “*σ*_i_” (eqn (24)) values for the reported samples, as expressed in Table 1.

“*B*” values change from 21.4 to 123.8 for the reported samples. “*B*” depends on polymer strength, CNT size, “*τ*_c_” and “*τ*” based on eqn (22). Therefore, it is expected to obtain the dissimilar levels of “*B*” for the reported samples. The highest and the lowest levels of “*B*” are obtained for polysilsesquioxane/MWCNT and PAN/MWCNT samples, respectively. The values of “*σ*_i_” are also calculated using eqn (24). “*σ*_i_” changes from 81.7 to 412.7 MPa for the samples. The strongest interphase is observed in chitosan-*g*-MWCNT sample, while polysilsesquioxane/MWCNT shows the poorest interphase among the samples. Since the interphase strength depends on polymer strength, “*B*”, CNT radius and interphase thickness, a high level of “*B*” may not produce a high interphase strength for the samples. However, it is observed that the lowest levels of “*τ*” and “*t*_eff_” for polysilsesquioxane/MWCNT sample cause the poorest interphase strength, while chitosan-*g*-MWCNT showing the highest levels of “*τ*” and “*t*_eff_” contains the strongest interphase. Accordingly, there is a direct correlation among “*τ*”, “*t*_eff_” and “*σ*_i_” parameters, which is reasonable, because all these parameters are a function of interfacial adhesion between polymer matrix and nanoparticles demonstrating the efficiency of interphase region for the stress transferring from polymer matrix to nanoparticles. The sensible correlations between these parameters validate the suggested equations in this study.

### 3.2 Analysis of parameters

In this section, the developed equations are used to investigate the influences of all parameters on the “*B*” (eqn (22)) and “*σ*_i_” (eqn (24)) terms. The accurate roles of all parameters in the mentioned terms approve the developed equations. Contour plots are used to calculate an output at different ranges of two parameters and average values of other factors as *σ*_m_ = 30 MPa, *σ*_f_ = 30 GPa, *R* = 10 nm, *u* = 1.2, *l* = 10 μm, *t* = 5 nm and *τ* = 200 MPa. The contour plots are useful to optimize the values of parameters producing the highest “*B*” and the strongest interphase in nanocomposites.

**Fig. 4:**
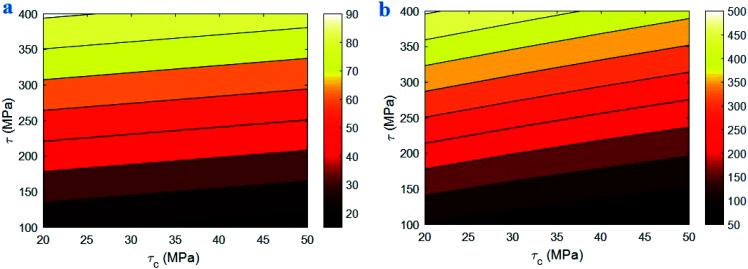
The calculations of (a) “*B*” and (b) interphase strength at different ranges of “*τ*_c_” and “*τ*” parameters using the developed equations.

Fig. 4 exhibits the impacts of “*τ*_c_” and “*τ*” parameters on the “*B*” and interphase strength according to the developed equations. The highest level of “*B*” is calculated as 90 at *τ*_c_ = 20 MPa and *τ* = 400 MPa, while “*B*” reduces to 10 at *τ*_c_ > 30 MPa and *τ* = 100 MPa. Additionally, *τ*_c_ > 35 MPa and *τ* < 130 MPa cause the interphase strength of 50 MPa, but *τ*_c_ = 20 MPa and *τ* = 400 MPa increase the interphase strength to 500 MPa. As a result, both plots display the same roles of “*τ*_c_” and “*τ*” parameters in the “*B*” and interphase strength. They indicate that a low critical interfacial shear strength and a high interfacial shear strength are optimum to obtain the high levels of “*B*” and interphase strength. On the other hand, high critical interfacial shear strength and poor interfacial shear strength weaken the “*B*” and interphase strength at the same time.

A poor “*τ*_c_” and a high “*τ*” grow the efficiency of interphase region for stress transferring, because they grow the effective interphase thickness (eqn (6)). In fact, a low “*τ*_c_” and a high “*τ*” enlarge the zone 2 in the interphase region (Fig. 1) strengthening the interphase for good stress transportation between polymer matrix and nanoparticles. However, high “*τ*_c_” and poor “*τ*” expand the zone 1 in the interphase region fading the interphase and causing the thin effective interphase. These explanations demonstrate that “*τ*” directly manipulates the effectiveness of interphase region, while “*τ*_c_” has an adverse role in the interphase properties. Since both “*B*” and interphase strength directly correlate to the interphase efficiency, “*τ*_c_” and “*τ*” reasonably control the mentioned terms validating the developed equations. Generally, a high level for *τ* − *τ*_c_ enhances the interphase characteristic in nanocomposites.

**Fig. 5:**
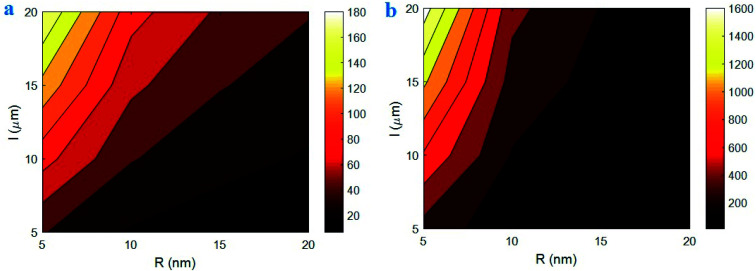
(a) “*B*” and (b) interphase strength as a function of “*R*” and “*l*” parameters by the developed equations.

Fig. 5 reveals the variation of “*B*” and interphase strength at different ranges of “*R*” and “*l*” parameters based on the developed equations. *R* > 15 nm and *l* < 15 μm fall the “*B*” to about 10, whereas the “*B*” significantly grows to 180 at *R* = 5 nm and *l* = 20 μm. Moreover, *R* > 15 nm largely decreases the interphase strength to about 0, but *R* = 5 nm and *l* = 20 μm produce the maximum interphase strength of 1600 MPa. Therefore, thin and large CNT optimize the values of “*B*” and interphase strength in the nanocomposites, while thick and short CNT weaken the interphase properties. Also, only thick CNT are enough to fail the interphase strength in nanocomposites. The large variation of “*B*” and interphase strength at different ranges of CNT size demonstrates that the CNT size highly manipulates the interphase properties.

Thin and long CNT produce a big surface area, which largely involves the surrounding polymer matrix. It means that thin and large CNT cause a big interfacial area between polymer matrix and nanoparticles. In this condition, the strong interfacial interaction exists between polymer matrix and CNT enhancing the “*B*” and interphase strength. However, thick and short CNT minimize the surface area of nanoparticles, which ineffectively involves the polymer matrix near the CNT. In this state, the nanoparticles slightly affect the surrounding polymer chains creating a thin and poor interphase in the nanocomposites. Moreover, the concentration of interphase region only depends on the CNT radius according to eqn (7). As a result, only thick CNT reduce the efficiency of interphase region in the nanocomposites. These observations approve the calculations of the developed equations for “*B*” and interphase strength at different levels of “*R*” and “*l*” parameters.

**Fig. 6:**
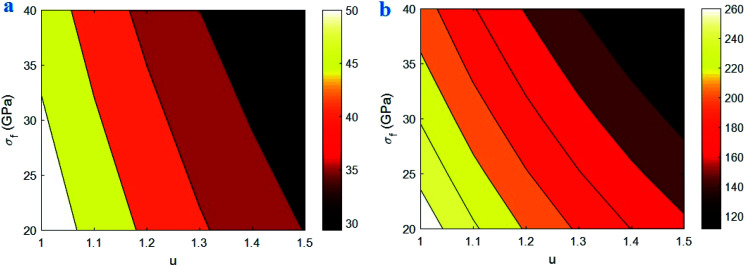
The calculations of developed equations for (a) “*B*” and (b) interphase strength at different ranges of “*u*” and “*σ*_f_” parameters.

Fig. 6 shows the influences of “*u*” and “*σ*_f_” parameters on the “*B*” and interphase strength based on the developed equations. The high levels of “*u*” and “*σ*_f_” decrease the “*B*” and interphase strength, but a high “*B*” and a strong interphase are obtained by the minimum levels of both “*u*” and “*σ*_f_” parameters. Therefore, the waviness and strength of CNT negatively affect the “*B*” and interphase strength in nanocomposites. However, both “*B*” and interphase strength slightly change at different ranges of CNT waviness and strength. Accordingly, these parameters insignificantly manipulate the interphase properties in the nanocomposites.

The waviness significantly worsens the effective length of CNT, which negatively influences the surface area of nanoparticles. In fact, a high waviness weakens the efficiency of CNT surface area at the interfacial area between polymer matrix and CNT. So, the waviness adversely affects the interphase properties such as “*B*” and “*σ*_i_” in the nanocomposites. On the other hand, a low waviness produces the efficient interfacial area between polymer matrix and CNT growing the interphase performance.

A high CNT strength raises the “*τ*_c_” (eqn (1)) diminishing the effective interphase thickness based on eqn (6). In other words, a high “*τ*_c_” worsens the effectiveness of interphase region in nanocomposites. Therefore, it is expected to observe the poor interphase properties at high CNT strength. However, a poor strength of CNT falls the “*τ*_c_” growing the stress bearing of interphase region and the effective interphase thickness. Accordingly, both CNT waviness and strength adversely affect the interphase properties confirming the developed equations. It should be noted that the strengthening efficiency of nanoparticles in nanocomposites correlates to their strength,^46^ although the strength of CNT differently controls the interphase properties. In can be concluded that the CNT strength should be optimized to provide a strong interphase and an effective strengthening in nanocomposites.

**Fig. 7:**
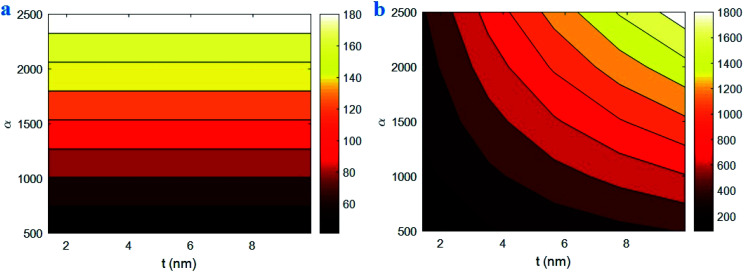
Variations of (a) “*B*” and (b) interphase strength at different ranges of “*t*” and “*α*” parameters by the developed equations.

Fig. 7 highlights the roles of “*t*” and “*α*” parameters in the interphase parameters using the developed equations. The “*B*” value of 180 is achieved by *α* > 2300, but *α* < 700 mainly decrease the “*B*” to 40. However, the various levels of “*t*” parameter do not change the “*B*”. Therefore, CNT aspect ratio directly manages the “*B*”, while the interphase thickness is an ineffective parameter. Furthermore, the interphase strength of 1800 MPa is obtained at *t* = 10 nm and *α* = 2500, but the interphase strength weakens to about 100 MPa at *t* < 2 nm and *α* < 1000. These results demonstrate that both CNT aspect ratio and interphase thickness directly influence the interphase strength in nanocomposites.

According to eqn (22), “*B*” depends on “*τ*”, “*τ*_c_”, polymer strength and CNT aspect ratio. Also, “*τ*_c_” correlates to CNT size and strength. As a result, the interphase thickness is an ineffective parameter on the “*B*” term. However, the thickness and strength of interphase region in nanocomposites depends on the interfacial adhesion between polymer matrix and nanoparticles.^55,56^ In other words, the strong interfacial interaction between polymer matrix and nanoparticles produces the thick and strong interphase. Therefore, the interphase strength directly depends on the interphase thickness approving the calculations of developed equations. Additionally, a high aspect ratio of CNT decreases the extent of “*τ*_c_” (eqn (1)), which positively manipulates the effective interphase thickness. Actually, the high-aspect-ratio CNT producing the strong interphase region positively influence the properties of polymer matrix.^63,64^ However, a low aspect ratio of CNT deteriorates the properties of interphase regions surrounding CNT. So, the aspect ratio directly handles the “*B*” and interphase strength confirming the developed equations.

## 4 Conclusions

“*B*” interphase term in Pukanszky model and interphase strength were expressed using “*τ*_c_” and “*τ*” by joining the suggested model and the developed Pukanszky model for tensile strength of nanocomposites. The experimental results of various samples confirmed the models and the parametric examinations validated the developed equations for “*B*” and interphase strength. A low critical interfacial shear strength and a high interfacial shear strength increased the “*B*” and interphase strength. Moreover, thin and large CNT optimized the “*B*” and interphase strength, while thick and short CNT weakened the interphase properties. In addition, only thick CNT lonely failed the interphase strength in nanocomposites. The different ranges of CNT size changed the “*B*” from 10 to 180 and the interphase strength from about 0 to 1600 MPa. These calculations demonstrated that the CNT size extremely manipulates the “*B*” and interphase strength among the parameters. The waviness and strength of CNT negatively affected the “*B*” and interphase strength. “*B*” changed from 10 to 90 and the interphase strength varied from 50 to 500 MPa at various values of waviness and strength of CNT. So, both “*B*” and interphase strength marginally changed at different ranges of these factors. CNT aspect ratio directly managed the “*B*”, while the interphase thickness played an ineffective role. However, high CNT aspect ratio and thick interphase positively handled the interphase strength.

## Conflicts of interest

There are no conflicts to declare.

## Supplementary Material

## References

[cit1] Hussein M. A., Abu-Zied B. M., Asiri A. M. (2018). Fabrication of EPYR/GNP/MWCNT carbon-based composite materials for promoted epoxy coating performance. RSC Adv..

[cit2] Wei L., Fu X., Luo M., Xie Z., Huang C., Zhou J., Zhu Y., Huang G., Wu J. (2018). Synergistic effect of CB and GO/CNT hybrid fillers on the mechanical properties and fatigue behavior of NR composites. RSC Adv..

[cit3] Liu C., Sergeichev I., Akhatov I., Lafdi K. (2018). CNT and polyaniline based sensors for the detection of acid penetration in polymer composite. Compos. Sci. Technol..

[cit4] Hu Y., Tang P., Li L., Yang J., Jian X., Bin Y. (2019). High absorption shielding material of poly(phthalazinone etherketone)/multiwall carbon nanotube composite films with sandwich configurations. RSC Adv..

[cit5] Saber O., Aljaafari A., Alshoaibi A., Osama A. (2019). A low-temperature technique and new strategy for the dual growth of carbon nanotubes and nanorods through the confinement of explosive materials inside a porous structure. RSC Adv..

[cit6] Shi S., Chen Y., Jing J., Yang L. (2019). Preparation and 3D-printing of highly conductive polylactic acid/carbon nanotube nanocomposites *via* local enrichment strategy. RSC Adv..

[cit7] Wang H., Li Z., Hong K., Chen M., Qiao Z., Yuan Z., Wang Z. (2019). Property improvement of multi-walled carbon nanotubes/polypropylene composites with high filler loading *via* interfacial modification. RSC Adv..

[cit8] Silakaew K., Thongbai P. (2019). Significantly improved dielectric properties of multiwall carbon nanotube-BaTiO_3_/PVDF polymer composites by tuning the particle size of the ceramic filler. RSC Adv..

[cit9] Zare Y., Rhee K. Y. (2019). Following the morphological and thermal properties of PLA/PEO blends containing carbon nanotubes (CNTs) during hydrolytic degradation. Composites, Part B.

[cit10] Zare Y., Rhee K. Y. (2019). The effective conductivity of polymer carbon nanotubes (CNT) nanocomposites. J. Phys. Chem. Solids.

[cit11] Mazov I., Burmistrov I., Il'inykh I., Stepashkin A., Kuznetsov D., Issi J. P. (2015). Anisotropic thermal conductivity of polypropylene composites filled with carbon fibers and multiwall carbon nanotubes. Polym. Compos..

[cit12] Russell R. A., Foster L. J. R., Holden P. J. (2018). Carbon nanotube mediated miscibility of polyhydroxyalkanoate blends and chemical imaging using deuterium-labelled poly(3-hydroxyoctanoate). Eur. Polym. J..

[cit13] Zare Y., Rhee K. Y., Park S.-J. (2019). A developed equation for electrical conductivity of polymer carbon nanotubes (CNT) nanocomposites based on Halpin–Tsai model. Results Phys..

[cit14] Zare Y., Rhee K. Y. (2019). Effects of interphase regions and filler networks on the viscosity of PLA/PEO/carbon nanotubes biosensor. Polym. Compos..

[cit15] Rostami A., Eskandari F., Masoomi M., Nowrouzi M. (2019). Evolution of Microstructure and Physical Properties of PMMA/MWCNTs Nanocomposites upon the Addition of Organoclay. Journal of Oil, Gas and Petrochemical Technology.

[cit16] Rostami A., Vahdati M., Alimoradi Y., Karimi M., Nazockdast H. (2018). Rheology provides insight into flow induced nano-structural breakdown and its recovery effect on crystallization of single and hybrid carbon nanofiller filled poly(lactic acid). Polymer.

[cit17] Rostami A., Vahdati M., Nazockdast H. (2018). Unraveling the localization behavior of MWCNTs in binary polymer blends using thermodynamics and viscoelastic approaches. Polym. Compos..

[cit18] Zhou Z., Sarafbidabad M., Zare Y., Rhee K. Y. (2018). Prediction of storage modulus in solid-like poly(lactic acid)/poly(ethylene oxide)/carbon nanotubes nanocomposites assuming the contributions of nanoparticles and interphase regions in the networks. J. Mech. Behav. Biomed. Mater..

[cit19] Zare Y., Rhee K. Y., Park S. J. (2019). Simple model for hydrolytic degradation of poly(lactic acid)/poly(ethylene oxide)/carbon nanotubes nanobiosensor in neutral phosphate-buffered saline solution. J. Biomed. Mater. Res., Part A.

[cit20] Hassanzadeh-Aghdam M. K., Ansari R., Mahmoodi M. J. (2019). Thermo-mechanical properties of shape memory polymer nanocomposites reinforced by carbon nanotubes. Mech. Mater..

[cit21] Hasanzadeh M., Ansari R., Hassanzadeh-Aghdam M. (2019). Evaluation of effective properties of piezoelectric hybrid composites containing carbon nanotubes. Mech. Mater..

[cit22] Syrgiannis Z., Trautwein G., Calvaresi M., Modugno G., Zerbetto F., Carraro M., Prato M., Bonchio M. (2019). Controlling Size-Dispersion of Single Walled Carbon Nanotubes by Interaction with Polyoxometalates Armed with a Tryptophan Tweezer. Eur. J. Inorg. Chem..

[cit23] Montazeri A., Chitsazzadeh M., Azad R., Madah D. (2017). The Dispersion Effect of Carbon Nanotubes on the Viscoelastic Properties of Epoxy by Perez Model. Int. J. Chemoinf. Chem. Eng..

[cit24] Rostami A., Moosavi M. I. (2019). High-performance thermoplastic polyurethane nanocomposites induced by hybrid application of functionalized graphene and carbon nanotubes. J. Appl. Polym. Sci..

[cit25] Ebrahimi H., Roghani-Mamaqani H., Salami-Kalajahi M., Shahi S., Abdollahi A. (2018). Preparation of Furfuryl Alcohol-Functionalized Carbon Nanotube and Epoxidized Novolac Resin Composites with High Char Yield. Polym. Compos..

[cit26] Esbati A., Irani S. (2018). Effect of functionalized process and CNTs aggregation on fracture mechanism and mechanical properties of polymer nanocomposite. Mech. Mater..

[cit27] Kalkhoran A. H. Z., Naghib S. M., Vahidi O., Rahmanian M. (2018). Synthesis and characterization of graphene-grafted gelatin nanocomposite hydrogels as emerging drug delivery systems. Biomed. Phys. Eng. Express.

[cit28] Naghib S. M. (2019). Two dimensional functionalized methacrylated graphene oxide nanosheets as simple and inexpensive electrodes for biosensing applications. Micro Nano Lett..

[cit29] Mamaghani K. R., Naghib S. M., Zahedi A., Mozafari M. (2018). Synthesis and microstructural characterization of GelMa/PEGDA hybrid hydrogel containing graphene oxide for biomedical purposes. Mater. Today: Proc..

[cit30] Naghib S. M. (2016). Fabrication of Nafion/Silver Nanoparticles/Reduced Graphene Nanosheets/Glucose Oxidase Nanobiocomposite for Electrochemical Glucose Biosensing. Anal. Bioanal. Electrochem..

[cit31] Seyfoori A., Ebrahimi S. S., Omidian S., Naghib S. M. (2019). Multifunctional magnetic ZnFe_2_O_4_-hydroxyapatite nanocomposite particles for local anti-cancer drug delivery and bacterial infection inhibition: an *in vitro* study. J. Taiwan Inst. Chem. Eng..

[cit32] Rajaee P., Ghasemi F. A., Fasihi M., Saberian M. (2019). Effect of styrene-butadiene rubber and fumed silica nano-filler on the microstructure and mechanical properties of glass fiber reinforced unsaturated polyester resin. Composites, Part B.

[cit33] Ajorloo M., Fasihi M., Ohshima M., Taki K. (2019). How are the thermal properties of polypropylene/graphene nanoplatelet composites affected by polymer chain configuration and size of nanofiller?. Mater. Des..

[cit34] Ajorloo M., Fasihi M., Khoramishad H. (2020). The role of nanofiller size and polymer chain configuration on the properties of polypropylene/graphite nanoplates composites. J. Taiwan Inst. Chem. Eng..

[cit35] Fasihi M., Abolghasemi M. R. (2012). Oxygen barrier and mechanical properties of masterbatch-based PA6/nanoclay composite films. J. Appl. Polym. Sci..

[cit36] Zare Y., Rhee K. Y., Park S.-J. (2019). A modeling methodology to investigate the effect of interfacial adhesion on the yield strength of MMT reinforced nanocomposites. J. Ind. Eng. Chem..

[cit37] Zare Y., Garmabi H., Rhee K. Y. (2018). Prediction of complex modulus in phase-separated poly(lactic acid)/poly(ethylene oxide)/carbon nanotubes nanocomposites. Polym. Test..

[cit38] Peng W., Rhim S., Zare Y., Rhee K. Y. (2019). Effect of “*Z*” factor for strength of interphase layers on the tensile strength of polymer nanocomposites. Polym. Compos..

[cit39] Hassanzadeh-Aghdam M. K., Mahmoodi M. J., Ansari R. (2019). Creep performance of CNT polymer nanocomposites-An emphasis on viscoelastic interphase and CNT agglomeration. Composites, Part B.

[cit40] Rafiee R., Pourazizi R. (2015). Influence of CNT functionalization on the interphase region between CNT and polymer. Comput. Mater. Sci..

[cit41] Montazeri A., Naghdabadi R. (2010). Investigation of the interphase effects on the mechanical behavior of carbon nanotube polymer composites by multiscale modeling. J. Appl. Polym. Sci..

[cit42] Zare Y., Rhee K. Y. (2019). Evaluation of the Tensile Strength in Carbon Nanotube-Reinforced Nanocomposites Using the Expanded Takayanagi Model. JOM.

[cit43] Razavi R., Zare Y., Rhee K. Y. (2019). The roles of interphase and filler dimensions in the properties of tunneling spaces between CNT in polymer nanocomposites. Polym. Compos..

[cit44] Zare Y., Rhee K. Y., Park S.-J. (2019). Modeling the roles of carbon nanotubes and interphase dimensions in the conductivity of nanocomposites. Results Phys..

[cit45] Zare Y., Rhee K. Y. (2020). Tensile modulus prediction of carbon nanotubes-reinforced nanocomposites by a combined model for dispersion and networking of nanoparticles. J. Mater. Res. Technol..

[cit46] Razavi R., Zare Y., Rhee K. Y. (2018). A model for tensile strength of polymer/carbon nanotubes nanocomposites assuming the percolation of interphase regions. Colloids Surf., A.

[cit47] Amraei J., Jam J. E., Arab B., Firouz-Abadi R. D. (2019). Modeling the interphase region in carbon nanotube-reinforced polymer nanocomposites. Polym. Compos..

[cit48] Jamalzadeh N., Heidary S., Zare Y., Rhee K. Y. (2018). A multistep methodology based on developed Takayanagi, Paul and Ouali models for tensile modulus of polymer/carbon nanotubes nanocomposites above percolation threshold assuming the contribution of interphase regions. Polym. Test..

[cit49] Lazzeri A., Phuong V. T. (2014). Dependence of the Pukánszky’s interaction parameter *B* on the interface shear strength (IFSS) of nanofiller-and short fiber-reinforced polymer composites. Compos. Sci. Technol..

[cit50] Rafiee R. (2013). Influence of carbon nanotube waviness on the stiffness reduction of CNT/polymer composites. Compos. Struct..

[cit51] Zare Y., Rhee K. Y. (2019). A multistep methodology for effective conductivity of carbon nanotubes reinforced nanocomposites. J. Alloys Compd..

[cit52] Zare Y. (2015). Effects of interphase on tensile strength of polymer/CNT nanocomposites by Kelly–Tyson theory. Mech. Mater..

[cit53] Kolařík J. (1997). Three-dimensional models for predicting the modulus and yield strength of polymer blends, foams, and particulate composites. Polym. Compos..

[cit54] Pukanszky B. (1990). Influence of interface interaction on the ultimate tensile properties of polymer composites. Composites.

[cit55] Li D., Liu Q., Yu L., Li X., Zhang Z. (2009). Correlation between interfacial interactions and mechanical properties of PA-6 doped with surface-capped nano-silica. Appl. Surf. Sci..

[cit56] Choi H. K., Yu J., Kim E., Shin E. S. (2018). Estimation of interfacial properties of nanocomposites using an analytical interphase model. Compos. Struct..

[cit57] Ashraf M. A., Peng W., Zare Y., Rhee K. Y. (2018). Effects of size and aggregation/agglomeration of nanoparticles on the interfacial/interphase properties and tensile
strength of polymer nanocomposites. Nanoscale Res. Lett..

[cit58] Zare Y., Rhee K. Y. (2019). A Simulation Work for the Influences of Aggregation/Agglomeration of Clay Layers on the Tensile Properties of Nanocomposites. JOM.

[cit59] Yuen S. M., Ma C. C. M. (2008). Morphological, electrical, and mechanical properties of multiwall carbon nanotube/polysilsesquioxane composite. J. Appl. Polym. Sci..

[cit60] Cao X., Dong H., Li C. M., Lucia L. A. (2009). The enhanced mechanical properties of a covalently bound chitosan-multiwalled carbon nanotube nanocomposite. J. Appl. Polym. Sci..

[cit61] Ji J., Sui G., Yu Y., Liu Y., Lin Y., Du Z., Ryu S., Yang X. (2009). Significant improvement of mechanical properties observed in highly aligned carbon-nanotube-reinforced nanofibers. J. Phys. Chem. C.

[cit62] Liu Y.-L., Chen W.-H., Chang Y.-H. (2009). Preparation and properties of chitosan/carbon nanotube nanocomposites using poly(styrene sulfonic acid)-modified CNTs. Carbohydr. Polym..

[cit63] Zare Y., Rhee K. Y. (2018). A multistep methodology for calculation of the tensile modulus in polymer/carbon nanotube nanocomposites above the percolation threshold based on the modified rule of mixtures. RSC Adv..

[cit64] Chen S., Sarafbidabad M., Zare Y., Rhee K. Y. (2018). Estimation of the tensile modulus of polymer carbon nanotube nanocomposites containing filler networks and interphase regions by development of the Kolarik model. RSC Adv..

